# The Hyperproduction of Polyhydroxybutyrate Using *Bacillus mycoides* ICRI89 through Enzymatic Hydrolysis of Affordable Cardboard

**DOI:** 10.3390/polym14142810

**Published:** 2022-07-10

**Authors:** Fady Abdelmalek, Alexander Steinbüchel, Marian Rofeal

**Affiliations:** 1 International Center for Research on Innovative Biobased Materials (ICRI-BioM)-International Research Agenda, Lodz University of Technology, Zeromskiego 116, 90-924 Lodz, Poland; fady.abdelmalek@p.lodz.pl (F.A.); alexander.steinbuchel@p.lodz.pl (A.S.); 2 Department of Botany and Microbiology, Faculty of Science, Alexandria University, Moharam Bek, Alexandria 21521, Egypt

**Keywords:** polyhydroxyalkanoates, poly(3-hydroxybutrate), enzymatic hydrolysis, lignocellulosic waste, cardboard waste, waste management, *Bacillus* sp.

## Abstract

Bioplastics are contemplated as remarkable substitutes for conventional plastics to accommodate green technological advancements. However, their industrial production has not been fully implemented owing to the cost of carbon resources. From another perspective, valorizing different paper mill wastes has become a prominent research topic. These materials may serve as an affording sustainable feedstock for bioplastic production. Adjustment of cardboard waste hydrolysate as suitable fermentation media for production of bacterial polyhydroxyalkanoates (PHAs) has been investigated. Cardboard samples were defibered and dried before enzymatic hydrolysis. The enzymatic degradation of commercial cellulase was monitored over 15 days. Interestingly, 18.2 ± 0.2 g/L glucose yield was obtained from 50 g cardboard samples using a 1.5% (*v*/*v*) enzyme concentration. The samples exhibited maximum weight loss values of 69–73%. Meanwhile, five soil samples were collected from local sites in Lodz, Poland. A total of 31 bacterial isolates were screened and cultured on Nile blue plates. Analysis of the 16S rRNA gene sequence of the most potent producer revealed 100% similarity to *Bacillus mycoides*. Cardboard hydrolysates whole medium, modified MSM with cardboard hydrolysate and nitrogen depleted MSM with cardboard hydrolysate were utilized for PHA production, followed by PHA productivity and cell dry weight (CDW) estimation compared to glucose as a standard carbon source. An impressive PHA accumulation of 56% CDW was attained when the waste hydrolysate was used as a carbon source. FTIR and NMR analysis of the isolated PHA indicated that functional groups of the polymer were related to PHB (polyhydroxybutyrate). Thermal analysis demonstrates that PHB and PHB-CB (PHB produced from cardboard hydrolysate) have degradation temperatures of 380 and 369 °C, respectively, which reflect the high thermal stability and heat resistance compared to the same properties for a standard polymer. This is the first demonstration of full saccharification of corrugated cardboard paper waste for high-level production of PHA. In addition, the attained PHB productivity is one of the highest levels achieved from a real lignocellulosic waste.

## 1. Introduction

It is acknowledged that there has been a growing need for bioplastics and biodegradable polymers in numerous fields owing to their diversified merits [1]. Polyesters are in high demanded in several sectors, such as pharmaceutical nanodelivery, food safety, and biomedical applications [2,3]. PHAs are efficient substitutes for petrochemical plastics from fossil fuels. They have been exploited in 3D printing inks, tissue engineering scaffolds, smart packaging materials, and biocompatible implants [4]. Moreover, modified PHA variants have become potent components as antimicrobial and antiviral agents, as well as targeting anticancer ligands [5,6]. Nevertheless, elevated production costs continue to inhibit scale-up. From an environmental perspective, various nations have urgent policies to turn to natural plastics manufacturing to mitigate the widespread usage of synthetic plastics [7]. Almost 4.8–12.7 million tons of plastic wastes are expected to enter the ocean by 2025, with no expected measures to manage this situation. PHAs are microbial polyesters accumulated inside bacterial cells under nitrogen limitations and excess carbon supplementation. The bioprocess technology for these polymers is still facing significant hindrance due to the negative effect of high-level production economics [8].

In this context, the usage of raw and waste materials has often been investigated in an attempt to identify economical strategies for bioplastic production. A strong example of this is a recent project which valorized marine algal biomass hydrolysate as promising feed stock for sustainable PHA synthesis. A simple acid pre-treatment for three marine algal biomass was performed in search of affordable sources of carbon to support microbial metabolism. A reasonable content of PHA was successfully produced using 3 different algal hydrolysates media from *Halomonas pacifica* ASL10 and *Halomonas salifodiane* ASL11 [9]. Since the issue of PHA commercialization has not been resolved, affordable alternative carbon sources, such as the use of agricultural waste [10], household waste [11], fats, oils, industrial by-products [12], glycerol, sugars, wastewater, and lignocellulosic materials [13], have been considered.

One of the most promising sustainable feedstocks for microbial polymer synthesis is lignocellulosic materials. Recently, a 70% yield of CDW (cell dry weight) poly(3-hydroxybutyrate) (P(3HB)) was obtained using *Burkholderia sacchari* DSM 17165 to promote enzymatically hydrolyzed wheat straw as a substitute medium [14]. Pretreatment of rice straw with dilute aqueous acid as an abundant sugar resource to provide a carbon source for producing high-value products, such as PHB using *B. megaterium* [15]. Mechanical conversion of biomass into smaller particles and fibers is commonly used to disrupt the lignocellulosic matrix and to gain access to the carbohydrates. Chemical pretreatment is frequently used to assist lignin decomposition and removal [16]. These procedures can be expensive, and they constitute a major impediment to broad carbohydrate production from lignocellulosic sources. These simple sugars can be used for biofuel production or other goods, such as biodegradable and compostable plastics, after the carbohydrate polymers are broken down to monomers [14].

Among these materials, cardboard is a yet undiscovered substance for affordable glucose supplementation. It is mainly composed of cellulose 56.1%, hemicellulose 10.4%, and lignin 12.8% [17]. They have been successfully used to obtain fermentable sugars, such as glucose [18,19]. Cardboard and other lignocellulosic stocks might undergo pretreatment, such as alkaline or paper hydrolysis, to ameliorate the efficiency of the enzymatic hydrolysis of cellulose. However, owing to the potential risks related to the formation of toxic compounds in alkaline or acidic hydrolysis, commercial cellulases digestion could be the optimum and most economical approach for the generation of glucose or fermentable sugars [15].

*Bacillus* sp. Has been widely used in industry and in academia due to the stability of its replication and plasmid maintenance, as well as its importance and supremacy in PHA production [20]. Furthermore, *Bacillus* sp. has a significant advantage over other bacterial species to produce PHAs due to the absence of a lipopolysaccharide layer, which makes extraction easier, as well as its ability to grow in low-cost raw materials and a high growth rate compared to other bacteria [21]. *Bacillus* sp. metabolism has proven to be rich, generating high-value products, such as lipopeptides, biosurfactants, antiviral proteins, and enzymes [22,23]. Thus, thorough screening of possible *Bacillus* strains from stress-prone environments, improved PHA synthesis methodologies, and the addition of low-cost carbon sources may all contribute to make the entire process more cost-effective [20].

This study investigates a successful bioconversion of cardboard to one of the most important polyesters, PHB. The saccharification of cardboard samples was conducted via enzymatic hydrolysis. A set of experiments was performed to optimize the fermentation process of cardboard hydrolysate as a feedstock for a locally isolated *Bacillus* strain. This represents the first attempt to valorize cardboard as a reliable source of glucose for microbial PHAs synthesis.

## 2. Materials and Methods

### 2.1. Materials and Chemicals

A commercial cellulase enzyme NewCell Conc L (NewEnzymes, Portugal) was kindly provided by Professor Stanislaw Bielecki, Lodz University of Technology, Poland. The enzyme preparation was composed of Cellulase and, 2-Benzisothiazolin-3(2H)-one (>1%). Dinitrosalicylic acid (DNS) was purchased from Sigma-Aldrich, Germany. The glucose detecting kit was obtained from Biomaxima, Poland. All other chemicals and media components were purchased from Pol-Aura, Poland.

### 2.2. Cardboard Sample Preparation

Corrugated cardboard specimens were obtained from local public solid waste containers. The contents of cellulose, hemicellulose, and lignin were detected according to the Van Soest and Robertson method [24]. The specimens were cut into pieces before being suspended in water at 60 °C for 24 h at a solid concentration of 50 g/L and defibered for 1 min using an IKA T50 Ultra Turrax Mixer. Samples that had been defibered were filtered and air dried for further examination [25].

### 2.3. Enzymatic Degradation of Cardboard Samples

Enzymatic hydrolysis tests were conducted using a commercial cellulase enzyme preparation. The specific activity of 65 ± 0.3 FPU/mL and enzyme protein concentration of 122 ± 0.3 mg/mL were determined using filter paper activity and Bradford methods [26,27]. The hydrolysis process took place in Erlenmeyer flasks at 55 °C, 150 rpm. The pH was preserved at 5.5 using 0.05 N citric acid–sodium citrate buffer.

(a) Determination of optimum incubation time

To determine the optimum incubation time for the enzyme activity, 50 g samples of defibered cardboard were incubated with 1.5% (*v*/*v*) cellulase enzyme in 1 L at different time intervals (1–15 days). The released glucose concentrations were determined by calorimetric kit at 540 nm.

(b) Weight loss

Different sample weights (20, 50, 80, and 100 g) were used for enzyme activity assessment using different enzyme concentrations at 55 °C, 150 rpm for 7 days incubation. The remaining cardboard was filtrated, dried at 70 °C for 24 h and weighed [25]. The following equation was used to calculate the proportion of cardboard consumption:

(W0 − Wt) × 100/W0

where W0 is the sample’s dry weight at zero time and Wt is the sample’s dry weight after 7 days incubation.

(c) Determination of reducing sugars

The quantity of reducing sugars released in the enzyme-cardboard mixture was determined by DNS reagent technique [28,29]. A total of 1 g dinitro salicylic acid was mixed with 10 mL distilled water and agitated for 10 min. A volume of 500 µL supernatant was mixed with 500 µL dinitrosalicylic acid reagent, left for 15 min at 28 °C, boiled for 5 min, then rapidly cooled, and the optical density was measured at 540 nm.

(d) Determination of liberated glucose

The enzymatic hydrolysis processes were stopped after enzymatic hydrolysis by keeping the solution at 4 °C for 2 h. The supernatants were recovered by centrifugation at 4 °C for 10 min at 8000 g. The glucose colorimetric kit (Biomaxima, Poland) was used to determine the glucose content of the hydrolysis liquors [30].

### 2.4. Collection of Samples, Growth Conditions, and Screening of PHA Producing Isolates

Soil samples were collected from several places in Lodz, Poland. In brief, samples were collected in sterile containers and transported directly to the lab, where they were serially diluted and inoculated on PHA screening plates. The screening medium is a Linko medium containing 2% glucose and 0.02 mg/L Nile blue stain as an indicator (Sigma-Aldrich-Darmstadt, Germany). Under UV light, the PHA synthesizing colonies appear orange [31]. Pure cultures of PHA-producing bacteria were then grown on Nutrient agar plates (pH 7.0 at 37 °C for 24 h) for future usage. Sudan black-B (SBB) stain (Sigma-Aldrich-Darmstadt, Germany) was used to perform a confirmatory screening test for PHA generating isolates. The accessibility of the C/N ratio in the medium plays a key role in PHA production. The use of a high carbon-containing medium promotes PHA accumulation. Thus, the medium employed was previously reported by [31] with minor adjustments. The production medium contained (in g/L) glucose (20), (NH_4_)_2_SO_4_ (0.5), KH_2_PO_4_ (2.0), Na_2_HPO_4_ (2.0), MgSO_4_·7H_2_O (0.5), Na_2_HCO_3_ (0.5), and CaCl_2_ (0.01) and a 100 mL trace element solution containing ZnSO_4_·7H_2_O (0.01), MnCl_2_·4H_2_O (0.003), H_3_BO_4_ (0.003), CuCl_2_·2H_2_O (0.001), and NICI_2_·6H_2_O (0.002). The incubation was carried out at 37 °C, pH 7 and 160 rpm.

### 2.5. Identification and Characterization of PHA Producing Isolates

The Genomic Mini kit (A&A biotechnology, Pomeranian Voivodeship, Poland) was used to extract genomic DNA from the selected bacterial strain, with minor adjustments to the first step: bacterial cells were treated with lysozyme and incubated at 37 °C for 20 min. The PCR was carried out using a MJ Mini Gradient Thermal Cycler (Bio-Rad, Hercules, CA, USA). The 16S rRNA gene was amplified using universal primers 27F and 1492R (5′-AGAGTTTGATCCTGGCTCAG-3′ 5′-GGTTACCTTGTTACGACTT-3′). Each PCR reaction contained 40 pmol of each primer, 1.5 U of RedTaq ReadyMix DNA polymerase (Sigma-Aldrich, St. Louis, MO, USA) and 20 ng of template DNA, and it was built up to 50 µL with PCR grade water. A 1.0% (*w*/*v*) agarose gel electrophoresis in 0.5 TBE buffer was used to identify PCR products (Sigma-Aldrich). The Big Dye Terminator Ready Reaction Cycle Sequencing kit was used to purify PCR products and extract gene nucleotide sequences (Applied Biosystems, Foster City, CA, USA). The PCR products were examined using an Applied Biosystems model 3730 Genetic Analyzer. The nucleotide sequences of the 16S rRNA gene were proofread, assembled, and aligned in Vector NTI Express Software (Life Technologies, Thermo Fisher Scientific Inc., Waltham, MA, USA), and they were compared with sequences available in the National Center for Biotechnology Information (NCBI, Bethesda, MD, USA), using the BLASTN algorithm (Version 2.2.30+) [31].

### 2.6. PHA Production Extraction and Purification

(a) MSM supplemented with glucose as a carbon source

The isolated PHA producer was cultured in Nutrient broth overnight at 37 °C and 160 rpm before being transferred to Mineral Salt Medium (MSM) to produce PHA [9]. The medium was supplemented with 20 g (2% *w*/*v*) glucose and 0.5 g (0.05% (*w*/*v*) (NH_4_)_2_SO_4_ to maintain a higher C/N ratio. The production cultures were incubated at 37 °C, 160 rpm for 7 days.

(b) Cardboard hydrolysate as a whole medium

The production media were prepared by filtrating 1 L of the cardboard hydrolysate medium to remove the cardboard residues. The filtrate was then kept at 4 °C for 2 h to stop the enzyme activity. The filtrate medium was sterilized by filtration using 200 nm bacterial filters (Alchem, Poland), then inoculated with the PHA producing strain and incubated at 37 °C, 160 rpm for 7 days.

(c) Modified MSM with cardboard hydrolysate

PHA production was studied in a synthetic medium in which 1 L filtrate of cardboard hydrolysate was supplied with all components of MSM except glucose, where the cardboard hydrolysate would be the carbon source. The concentration of carbon source was adjusted to be almost 2% (*v*/*v*).

(d) Nitrogen depleted MSM with cardboard hydrolysate

The filtrate of cardboard hydrolysate (1 L) was supplied with all components of MSM except glucose and (NH_4_)_2_SO_4_, where the cardboard hydrolysate would be the source of carbon and nitrogen. The concentration of carbon source was adjusted to be almost 2% (*v*/*v*), as previously mentioned.

(e) Extraction of PHA

The whole volume of the fermentation medium was centrifuged at 4 °C in a cooling centrifuge (4500 rpm for 15 min), and the cell pellets were freeze dried. The dry pellet weight was measured, and the bacterial cell wall was disrupted by treating it with hot acetone (50 °C) for 20 min. The suspension was centrifuged at 4500 rpm for 15 min before being dried to eliminate excess acetone. PHA was dissolved with chloroform at 37 °C for 48 h, while shaking at 160 rpm. The precipitation of PHA was done using cold methanol and water (7:3) [31]. The percentage of PHA yield was determined by the following equation:

PHA yield% = W_PHA_/W_Cells_ * 100

where W_PHA_ resembles the weight of the polymer recovered from the freeze-dried cells weight (W_Cells_).

### 2.7. Characterization of the Produced Polymer

(a) Fourier Transform Infrared (FTIR)

The refined polymers were subjected to FTIR analysis. The study was carried out in a range of 400–4000 cm**^−^**^1^ using an FTIR Nicolet 6700 spectrophotometer and OMNIC 3.2 software (Thermo Scientific Products: Riviera Beach, FL, USA) [9].

(b) 1H and 13C NMR

Polymer samples (25 mg) were diluted in 1 mL deuterated chloroform (CDCl_3_) and analyzed using NMR. The investigation was carried out using a JEOL JNM ECA 500 MHz (JEOL, Japan) to determine the chemical structure of the produced polymers [31].

(c) TGA, DTG, and DTA

Thermal analysis was performed on the purified polymers using the DSC Q20 and TGA Q50 analyzers. The analysis was carried out in the presence of a 20 mL/min N_2_ flow. Using a sequential heating system (heating rate 10 °C/min, temperature range 0 to 600 °C), 10 mg of moisture-free distilled PHAs were heated. Following that, the polymer’s degradation temperature (*Td*), glass transition temperature (*Tg*), enthalpy of fusion (DH_m_), and melting temperature (*Tm*) were measured [31].

(d) XRD

The diffractograms were used to investigate the crystalline structure of the polyesters. A copper tube with a wavelength of 1.5418 A° was used to record the spectrum, which was operated at 30 kV and 10 mA. A 2 mm diameter capillary tube was employed, each scan was recorded in step-by-step mode from 0 to 100° (2θ) with 5° intervals, and the intensities were recorded [5].

### 2.8. Statistical Analysis

The statistically significant differences across the studies were examined using a one-way ANOVA with the Tukey test (*p* < 0.05 confidence). The process monitoring assays were performed in triplicate, and the results were reported as the mean value and its standard deviation. The data was analyzed using Prism 7 (GraphPad, Inc. San Diego, CA, USA).

## 3. Results and Discussion

### 3.1. Enzymatic Degradation of Cardboard Samples

**Figure 1:**
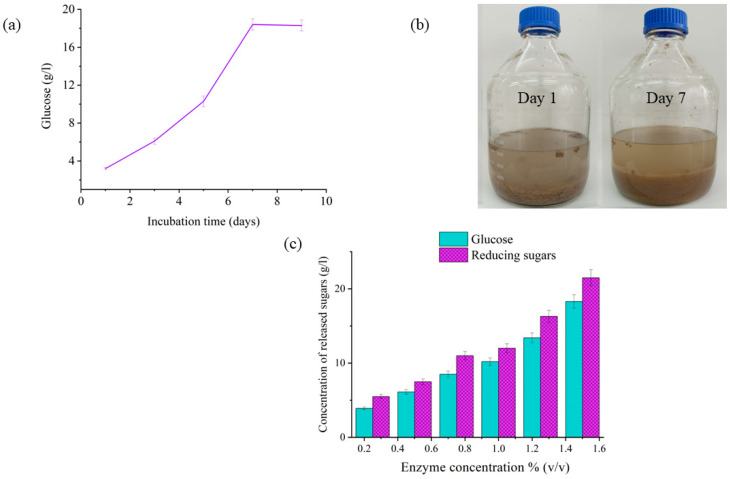
Enzymatic hydrolysis of cardboard, (**a**) Incubation time effect on the hydrolysis process, (**b**) presentation of the enzymatic hydrolysis process on cardboard fibers on the 1st and the 7th day and (**c**) colorimetric analysis of the enzymatic hydrolysis products, including reducing sugars and glucose.

Owing to the high percentage of cellulose in cardboard, which can reach up to 59% [25], it could be a promising source of carbon to produce value-added products. The complexity of polysaccharides in such waste (mainly cellulose and hemicellulose) has made cellulase systems paramount in liberating fermentable sugars. From an environmental perspective, the industrial implementation of the current study could assist in tackling waste accumulation issues in Europe, especially Poland. Paper and cardboard waste comprised approximately 15% of municipal solid waste from 2012 to 2017, whereas plastics resamples were 18%, and this data is expected to witness a considerable rise in the next years [32]. The chemical composition of corrugated cardboard showed that our samples contained 58.2% cellulose, 9.6% hemicellulose, 10.0% lignin, and 22.2% other components, which were in good line with another investigation [33]. Enzyme-assisted cardboard hydrolysis seems to be an appropriate approach for supplying PHA producers with sufficient amounts of carbon source in an economic manner. To assess cellulase activity on cardboard digestion, representative experimental concentration profiles of glucose and reducing sugars obtained in this set of experiments are shown in Figure 1.

Regarding the liberated glucose profile, the glucose concentration was directly proportional to incubation time till day 7 at pH 5.5 and 55 °C. Moreover, the highest released glucose concentration was reported to be 18.3 ± 0.2 g/L on day 7, which comprises approximately 62% of the total cellulose content in the investigated cardboard samples (Figure 1a), whereas it exhibited almost constant values from days 8 to 15 (data not shown). The reason for the positive correlation between the released glucose and incubation days could be attributed to cellulase activity on the cellulosic portion of cardboard [34]. Cellulases work synergistically to hydrolyze cellulose as it is degraded from crystalline or amorphous cellulose to small, soluble cellobiose fragments and finally to glucose [35]. The constant glucose records after day 7 demonstrated a complete saccharification of cellulose and hemicellulose contents, where the major morphological changes in the fiber structure took place. It might also be attributed to the depletion of cellulosic components in cardboard as well as the generation of metabolic by-products. (Figure 1b) [33]. Therefore, the incubation period of 7 days has been selected as the enzyme incubation time in the following experiments.

The effect of different enzyme concentrations was assessed on different samples’ weights in terms of cardboard degradation. Maximum weight loss values for all samples were observed at an enzyme concentration of 1.5% (*v*/*v*), which was found to be in the range of 69 to 73% (Table 1). The maximum weight loss values that resulted from the highest enzyme concentration supports the fact that cellulase catalyzes the polysaccharide decomposition by simply cleaving β-1,4-glycosidic linkages [36]. The enzymatic action of cellulase could be seen in Figure 1b, where the firm fibers of cardboard were transformed to powder-like structures after 7 days incubation at 55 °C.

The hydrolysis products, such as released glucose and reducing sugars, were determined in 50 g cardboard samples. The highest enzyme concentration (1.5% (*v*/*v*)) released 18.3 ± 0.3 g glucose, which was the maximum value compared with the lower cellulase concentrations. Moreover, the glucose percentage per 50 g cardboard sample was determined to be 36.5 ± 0.4%, which represents 18.3 ± 0.3 g glucose produced from a 50 g cardboard sample. This glucose content was estimated to be approximately 85.1 ± 0.1% of reducing sugars per 50 g sample (Figure 1c). Such data were consistent with the obtained glucose values measured at 36–63% (g/g of cellulose sample) using commercial enzymes cellulase CTec2 and hemicellulase HTec [33]. These results support the fact that the yielded carbon source would provide a sufficient supplement for PHA production.

### 3.2. Molecular Identification of the PHA Producer and Phylogenetic Analysis

**Figure 2:**
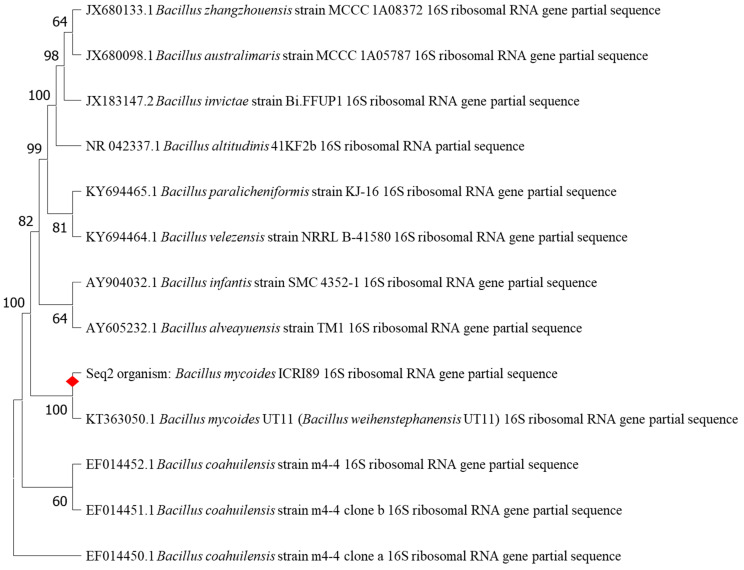
Neighbor-joining phylogenetic analysis based on 16S rRNA gene sequences showing the position of strain *B. mycoides* ICRI89. These phylogenetic relationships were identified by MEGA 11 sequence alignment editor (version 11.0.11).

The screened soil bacterial isolates resulted in 9 PHA producers, which were confirmed by orange color fluorescence on Nile blue plates and Sudan black staining. The bacterial isolate with the highest PHA productivity was selected for further molecular identification. The 16S rRNA study of the PHA producing strain exhibited a significant degree of similarity to the *Bacillus* sp. genera. A sequence comparison using BLAST showed strain ICRI89’s close relationship to *Bacillus mycoides* (formerly known as *Bacillus weihenstephanensis* UT11) with 100% similarity. The 16S rRNA sequence of the newly isolated strain was submitted to GenBank, NCBI as *Bacillus mycoides* ICRI89. A neighbor-joining dendrogram with several *Bacillus* sp. as an outer group shows the phylogentic position of *B. mycoides* ICRI89 (Figure 2).

### 3.3. Nucleotides Accession Numbers

Consensus sequences were generated with the MEGA 11 sequence alignment editor (version 11.0.11), and the sequences were then evaluated with the BLASTN software (NCBI) [37]. The 16S sequence was submitted to GenBank with the accession number ON231789.

### 3.4. MSM Supplemented with Glucose as a Carbon Source

**Figure 3:**
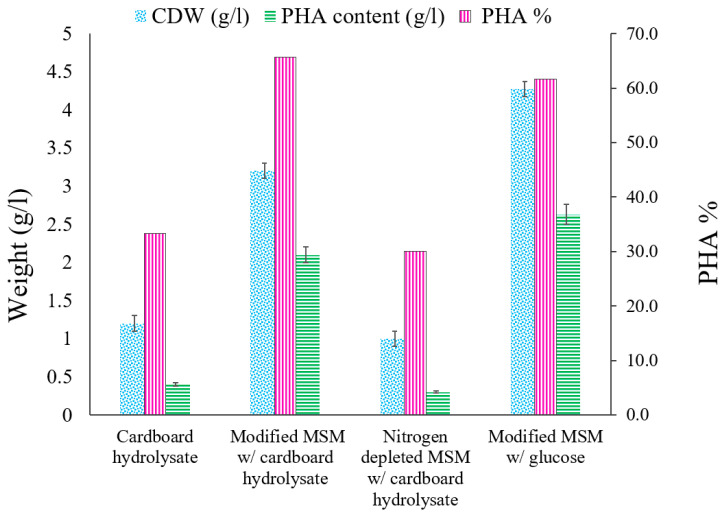
PHA content (g/L), cell dry weight (CDW) (g/L), and PHA productivity (%) in four different models.

*B. mycoides* ICRI89 was cultivated in 1 L MSM in 2 L flasks with 2% (*w*/*v*) glucose and 0.05% (*w*/*v*) (NH_4_)_2_SO_4_ at 37 °C, pH = 7, 160 rpm for 7 days. The biomasses from PHA production were collected, lyophilized, and weighed [31]. The lyophilized mass of *B. mycoides* ICRI89 was 4.27 g/L, and the generated polyester was recovered from the cells. The pure polymers produced were 2.63 ± 0.1 g/L, corresponding to 61.7% CDW (Figure 3). These results are highly similar to those of a previous investigation [38] in which *B. megaterium* accumulated a maximum PHA weight of 2.74 g/L with glucose as the sole carbon source. When compared to other carbon sources, such as arabinose, starch, lactose, lactic acid, glycerol, or sodium acetate, *Bacillus* sp. can efficiently metabolize glucose for greater PHA synthesis. As a result, when glucose was added as a carbon source, it produced the highest PHA content when compared to other carbon sources [38]. The maximum PHA yield in the current study was detected upon using (NH_4_)_2_SO_4_ as a nitrogen source. PHA synthesis was comparable when various nitrogen sources were used, including, with a minor difference, protease peptone, glycine, potassium nitrate, urea, and ammonium chloride. Previously, ammonium sulphate was shown to be the optimum nitrogen source for *B. mycoides* RLJ B017 [39] and *B. Megaterium* [40]. On the other hand, the current findings showed a higher PHA productivity than those of *Bacillus* sp. AZR-1, which used glucose for PHA production. *Bacillus* sp. AZR-1 produced CDW of 1.88 g/L with a PHA content up to 40%. It was shown to be extremely effective in exploiting soluble starch as a precursor for PHA accumulation, having a CDW of 0.87 g/L and a PHA content of 0.19 g/L, resembling 22% CDW. This amount is approximately half that when glucose was utilized as a carbon source, which is logical given that the starch hydrolysis process makes starch less useful when compared to simple monosaccharides, such as glucose [41]. This suggests that for growth and subsequent PHA synthesis, in general, these bacteria prefer simple monomers, such as glucose. Our isolate *B. mycoides* ICRI89 appears to have the most active metabolic machinery for synthesis of PHA. In many studies, there has been a positive correlation between the amount of glucose used as a carbon source and PHA production [42].

### 3.5. Cardboard Hydrolysate as a Whole Medium

Enzymatically hydrolyzed cardboard was employed as a complete medium to evaluate our new strain’s potential to accumulate PHA. When hydrolyzed cardboard was utilized as a PHA production medium for *B. mycoides* ICRI89, the highest PHA concentration was 0.4 ± 0.1 g/L, with 33.3% CDW PHA productivity (Figure 3). The PHA content witnessed an 84% reduction in comparison with that of glucose as a carbon source. In the same manner, there was an apparent decline in the yielded CDW to be 1.2 g/L compared to 4.27 g/L for the glucose standard medium. The major explanation for the low PHA concentration might be the lack of certain minerals and salts. Furthermore, the existence of KH_2_PO_4_ in the production medium at concentrations less than 0.1 g/L could reduce cellular proliferation, whilst concentrations higher than 0.1 g/L might improve PHA productivity. Thus, phosphate limitation (KH_2_PO_4_ and K_2_HPO_4_) was discovered to have an essential role in PHA build-up [43]. Enzymatically hydrolyzed waste paper was recently used as a sustainable feed stock for PHA synthesis by *B. Sacchari*. The bacterial strain accumulated 3.63 g/L PHA, which comprises approximately 44.2% CDW. The relatively higher yield compared to our study could be reasoned to performing enzymatic digestion for paper waste using an enzyme mixture of cellulase, β-glucosidase, and hemicellulose [44]. Such a mixture may have played a critical role in having a better saccharification of lignocellulosic biomass, including paper waste. That’s why it is recommended to examine the effect of different enzyme cocktails on the saccharification of different kinds of cardboard in future studies.

### 3.6. Modified MSM with Cardboard Hydrolysate

The filtrate of cardboard hydrolysate (1 L) was supplied with all components of MSM except glucose, where the cardboard hydrolysate would be the carbon source. The concentration of carbon source was adjusted to be almost 2% (*v*/*v*)**,** which represents the content of reducing sugar in cardboard hydrolysate. The PHA generating isolate *B. mycoides* ICRI89 had a maximum PHA concentration of 2.1 ± 0.1 g/L, which corresponds to 65.6% CDW (Figure 3). This experiment yielded almost the same PHA weights of that when glucose was used as the only carbon source. These findings are consistent with a previous study aiming at increasing PHA bioproduction using wheat straw lignocellulosic hydrolysates [14]. Since *B. sacchari* DSM 17165 can metabolize its primary carbohydrates, such as glucose, xylose, and arabinose, it was employed to generate PHA from wheat straw hydrolysates. When grown on a mixture of commercial C6 and C5 sugars as a control production medium, the *B. sacchari* cell weight of 6.0 g/L accumulated approximately 4.4 g/L PHA, resembling 70% CDW with a polymer on sugar yield of 0.18 g/g, whereas when wheat straw hydrolysates were used as the carbon source, these values reached approximately 4.4 g/L PHA, presenting 60% CDW [14]. Even though PHAs offer several environmental benefits, their high production costs limit their widespread application. One of the grand challenges facing these polymers scale-up is the high-cost feed stock, especially carbon sources, which comprise nearly the majority of the final cost [45,46]. Paper rejects, including cardboard, could be promising sources for essential and simple sugars for bacterial metabolism supporting polyester accumulation. Different cardboard rejects treatment procedures may provide more glucose and xylose per kilogram. For example, sodium hydroxide treatment of cardboard yielded approximately 373 g glucose and 61 g xylose per kilogram of rejects, which is regarded as an economical technique for PHA scale-up [47].

### 3.7. Nitrogen Depleted MSM with Cardboard Hydrolysate

Under nutrient-restricted environments, microbial polyesters accumulate with surplus carbon and polymerize as inclusion bodies [48]. To mimic such conditions, 1 L filtrate of cardboard hydrolysate was supplied with all components of MSM except glucose and (NH_4_)_2_SO_4_, where the cardboard hydrolysate would be the carbon and nitrogen sources. The concentration of carbon source was adjusted to be almost 2% (*v*/*v*), which represents the content of reducing sugar in cardboard hydrolysate. After 7 days of incubation at 37 °C and 160 rpm, *B. mycoides* ICRI89 used this hydrolysate to generate a PHA content of 0.3 ± 0.1 g/L (30% CDW) (Figure 3). The hydrolysate contains not only carbon but also nitrogen. The amount of nitrogen in the hydrolysate is insufficient to support biomass growth. A recent study [49] reported a low 0.003% nitrogen content in corrugated carboard acidic hydrolysate. Since high nitrogen concentrations directly increase cell biomass rather than PHA formation [50], PHA generation and cell proliferation were severely hindered.

When all the preceding data are compared, it is clear that the PHA concentration achieved by employing modified MSM with cardboard hydrolysate was extremely near to that obtained when glucose was used as the only carbon source. Sugar consumption in the cardboard hydrolysate medium was the primary cause of PHA production and build-up. Thus, polymers extracted from cells grown on glucose and cardboard hydrolysate medium as a carbon source were used for further characterization.

### 3.8. Analysis and Characterisation of the Purified Polymer

#### 3.8.1. FTIR

**Figure 4:**
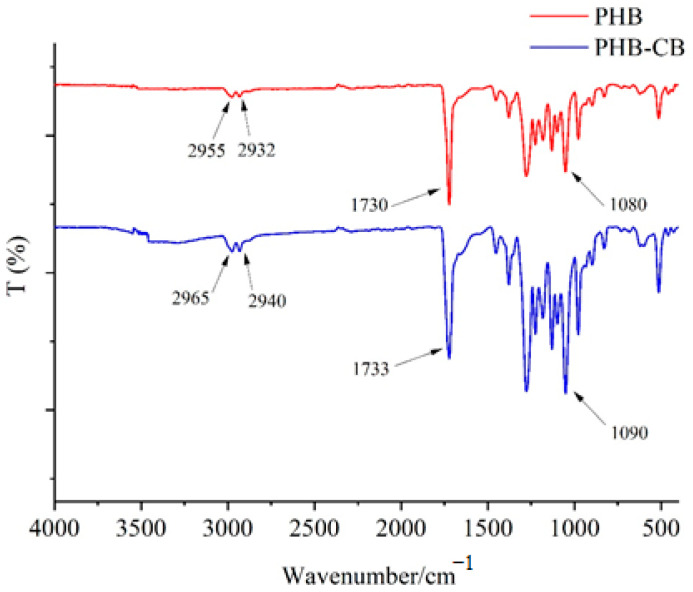
FTIR spectra of polymers isolated from *B. mycoides* ICRI89 grown in MSM containing either glucose (PHB) or cardboard hydrolysate (PHB-CB) as carbon sources.

The chemical structure of the purified biopolymers was determined using FTIR. The IR spectra of polymer generated in a medium containing glucose as a carbon source revealed three prominent absorption bands at 1730, 765, and 710 cm^−1^ due to ester, CH group, and carbonyl group, respectively. These bands also appeared in the IR spectra of the polyester produced from cardboard hydrolysate medium as a carbon source at 1733, 769, and 708 cm^−1^, respectively (Figure 4). The existence of -CH bonding is shown by the typical bands at 2955 and 2965 cm^−1^, while the C=O and ester groups are represented by the bands at 1730 and 1733 cm^−1^. The bands found at 1080 and 1090 cm^−1^ correspond to the C-O bonding for PHB derived from glucose and cardboard, respectively. The bands of the aforementioned polyester samples are quite similar to the bands of standard PHB, which affirms the high purity of the generated polymer. These findings are consistent with those reported by a recent study of PHB produced by *B. megaterium* MTCC 453 [51]. Because of the FTIR data, it is apparent both polymers produced from glucose (PHB) and cardboard hydrolysate (PHB-CB) as carbon sources are PHB, which is the common homopolymer of PHAs.

#### 3.8.2. NMR

**Figure 5:**
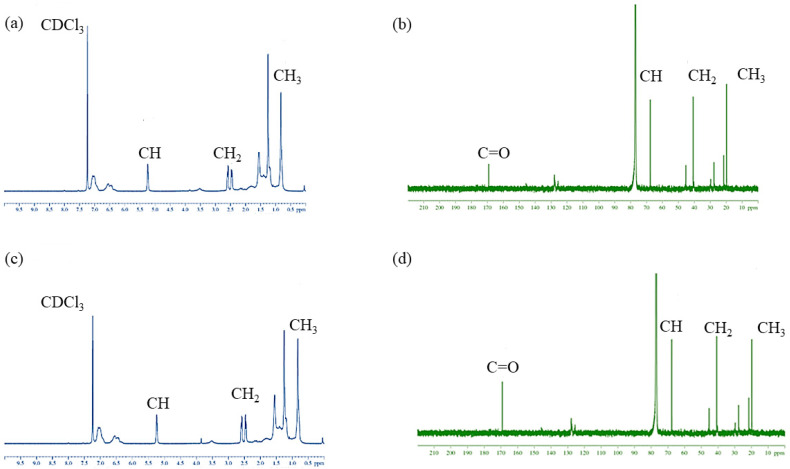
^1^HNMR and ^13^CNMR analysis of PHB and PHB-CB, (**a**) ^1^HNMR for PHB, (**b**) ^13^CNMR for PHB, (**c**) ^1^HNMR for PHB-CB and (**d**) ^13^CNMR for PHB-CB.

PHB samples were characterized using ^1^H NMR and ^13^C NMR spectroscopic methods. The ^1^H and ^13^C NMR spectra of PHB produced from *B. mycoides* ICRI89 by microbial fermentation of glucose and cardboard hydrolysate are shown in Figure 5. PHB characteristic peaks were detected in both PHB and PHB-CB, such as δ = 5.21 and 5.23 ppm, which correspond to –CH doublet, δ = 2.50 and 2.51 ppm for –CH_2_ multiplet, and δ = 1.21 and 1.22 of –CH_3_ doublet for the PHB and PHB-CB, respectively. The large peaks at δ = 7.3 ppm (Figure 5a,c) indicate the solvent (CD_3_Cl), while the small peaks at δ = 1.61 and 1.63 ppm are due to the minor H_-_O contamination of the solvent. These findings were identical to those obtained using the PHB standard. Hence, we determined that the polyesters produced by *B. mycoides* ICRI89 strain cultivated in glucose and cardboard hydrolysate as the carbon source were PHBs [52]. ^13^C NMR analysis also confirmed these findings. The functional groups C=O (170.5 and 170.3), CH (65.9 and 65.7 ppm), CH_2_ (42.55 and 41.9 ppm), and CH_3_ (19.5 and 19.6 ppm) peaks were assigned for PHB and PHB-CB, and they were similar to the PHB previously obtained from *Bacillus* sp. [53].

#### 3.8.3. TGA and DTG

**Figure 6:**
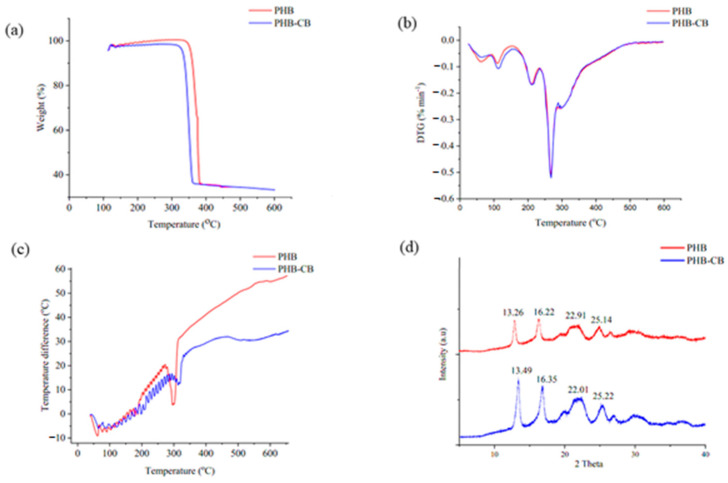
Characterization of PHB and PHB-CB demonstrating (**a**) TGA, (**b**) DTG, (**c**) DTA, and (**d**) XRD.

TGA profiles of PHB and PHB-CB synthesized by *B. mycoides* ICRI89 are depicted in Figure 6a. The TGA curve represents the weight loss of the synthesized PHB in two phases for the two generated polyesters generated. The first step of mass loss occurred at temperatures ranging from 100 to 180 °C. For PHB and PHB-CB, the mass loss was approximately 1.5 and 1.3% of total mass, respectively. This loss is caused by the evaporation of physically adsorbed solvents, such as methanol, chloroform, and others that have formed on the polymer surface. Furthermore, the second or major step of polymer degradation started after 200 °C, which occurs after the melting point of PHB. The decomposition process involves a molecular weight decrease, which includes chain scission and hydrolysis. The random chain scission process, which involves the breakage of C=O and C-O bonds in ester moieties by β-scission, destruction of crystalline areas, and depolymerization, is responsible for the rapid heat breakdown of PHB at this stage [54]. The second stage of weight loss occurred when the temperature increased further, as hydrolysis, chain scission, and the synthesis of crotonic acid all contribute to the deterioration process. From the analysis of the initial and the maximum degradation temperatures of main step weight loss and residual mass percentage for PHB and PHB-CB, the maximum degradation temperatures for PHB and PHB-CB were found to be 380 and 369 °C, respectively. As a result, it can be inferred that both forms of PHB exhibited greater thermal stability when compared to standard PHB, which was found to have a decomposition temperature of 285 °C [55]. Furthermore, the residual mass of PHB and PHB-CB is less than 1.5%. The second stage of degradation for PHB produced from *Bacillus* sp. ranges between 237 and 320 °C which is lower than our records. This implies that the synthesized PHBs have higher degrees of thermal stability than the PHB produced by *Bacillus* sp. N-2 [56].

The rate of mass loss of a polymer sample with relation to temperature was investigated using differential thermogravimetric (DTG) analysis (Figure 6b). The DTG curve peaks reflect the thermal stability of PHB in relation to the temperature at which the highest breakdown rate of the polymer matrix occurs. The DTG characteristic curves, as the TGA curves, revealed three distinct phases. The mass loss rate in the first phase was approximately 0.16 to 0.20 mass%/min until 190 to 200 °C, and the amount of residue is quite high in both PHB and PHB-CB samples. The maximum degradation temperature for PHB in the second stage of degradation was approximately 266 °C, with a maximum mass loss rate of 34%/min. PHB-CB’s maximum degradation temperature was approximately 268 °C, with a maximum mass loss rate of 32%/min. It has previously been reported that PHB standard has a degradation temperature of roughly 236 °C, with a maximum mass loss rate of 30%/min [51]. According to the results of the foregoing investigation, the PHB and PHB-CB produced by *B. mycoides* ICRI89 indicate strong thermal stability or resistance to heat deterioration.

#### 3.8.4. DTA

Differential Thermal Analysis (DTA) aids in determining breakdown heat (Figure 6c). This experiment was carried out to assess the cross-linking capabilities and the heat stability of the generated polymer. Due to the existence of cross-linking events during PHB degradation, an exothermic peak is found in the DTA thermogram. The curing temperatures, which are 331 and 325 °C for both PHB and PHB-CB, are the temperatures at which cross-linkage occurs. It is the most essential attribute that appears to be a major impediment to the commercial application of PHB, generating thermal instability due to a lack of cross-link capacity [57].

#### 3.8.5. XRD

The XRD spectra (Figure 6d) presents X-ray diffraction of PHB samples from both pure polyesters, PHB and PHB-CB. The observed peaks in XRD spectra for PHB are 2θ = 3.69°, 13.26°, 16.22°, 22.91°, and 25.14°, while the observed peaks in XRD spectra for PHB-CB are 2θ = 3.61°, 13.49°, 16.35°, 22.01°, and 25.22°. The peaks at 2θ = 13.26°,13.49°,16.22°, and 16.35°, which are the most intense and scattering peaks, each indicate an orthorhombic unit cell. The relatively weaker peaks observed at 2θ = 22.91° and 22.01° correspond to α-PHB crystal, while the minor spectra observed at 2θ = 25.14° and 25.22° denote PHB’s partly crystalline nature. The polymer matrix adopts a regular helicoidal shape with two antiparallel chains in the rhombic unit cell inside the crystalline domain [51,52,53,54,55,56,57,58]. Pradhan et al. [44] showed that the diffractogram of the produced PHBs in the current study is nearly equivalent to that of PHB produced from *Bacillus* sp. [51] Collectively, more efforts should be made to recycle environmental waste [59].

## 4. Conclusions

The current study proposes corrugated cardboard waste hydrolyzed by commercial cellulase as a cheap and readily available substrate for microbial PHB synthesis. This would remove one of the major barriers facing PHB scaling-up. The enzyme concentration affected the reducing sugars, reaching a satisfactory yield of 21.3 ± 0.1 g/L at an enzyme concentration of 1.5% (*v*/*v*) at 55 °C. Moreover, the newly isolated *B. mycoides* ICRI89 accumulated 2.1 ± 0.2 g/L of PHAs when grown on modified MSM containing cardboard hydrolysate, which was highly close to that produced when grown on MSM supplemented with glucose. It is important to note that the polymers purified from B. mycoides ICRI89 cells were almost entirely composed of PHB, according to FTIR, NMR, and XRD findings.

## 5. Future Prospects

The present research has several future prospects, including the industrial fermentation of cardboard and paper waste in humongous bioreactors, using several enzyme mixtures for promoting polyester synthesis. In addition, the methods of PHB purification from bacterial cells have to be improved to obtain pure polymer batches at a large scale.

## Figures and Tables

**Table 1:** Weight loss (%) of different cardboard samples after enzymatic hydrolysis. Mean and standard deviations were used to show the values. The presence of different superscript letters within the same column indicates significance (*p* < 0.05).

Cardboard Samples Weights (g)	100	80	50	20
Enzyme Concentration (%)	Weight Loss (%)			
0.25	20 ± 0.5 ^a^	23 ± 0.3 ^a^	26 ± 0.5 ^a^	30 ± 0.1 ^a^
0.5	32 ± 0.2 ^b^	33 ± 0.9 ^b^	36 ± 0.5 ^b^	39 ± 0.3 ^b^
0.75	51 ± 0.1 ^c^	50 ± 0.5 ^c^	50 ± 0.3 ^c^	52 ± 0.9 ^c^
1	60 ± 0.2 ^d^	61 ± 0.4 ^d^	64 ± 0.1 ^d^	66 ± 0.1 ^d^
1.25	65 ± 0.3 ^e^	67 ± 0.1 ^d^	68 ± 0.1 ^d^	70 ± 0.6 ^e^
1.5	69 ± 0.3 ^e^	69 ± 0.2 ^d^	70 ± 0.3 ^d^	73 ± 0.3 ^e^

## Data Availability

The accession number(s) can be found on the GenBank. Available online: https://www.ncbi.nlm.nih.gov/, ON231789 (Accessed on 17 May 2022).
